# Multi-task meta-initialized DQN for fast adaptation to unseen slicing tasks in O-RAN

**DOI:** 10.1371/journal.pone.0330226

**Published:** 2025-10-09

**Authors:** Bosen Zeng, Xianhua Niu

**Affiliations:** 1 Key Laboratory of Interior Layout Optimization and Security, Institutions of Higher Education of Sichuan Province, Chengdu Normal University, Chengdu, Sichuan, China; 2 Sichuan Provincial Key Laboratory of Education Digitalization Development and Evaluation, Chengdu Normal University, Chengdu, Sichuan, China; 3 School of Computer Science, Chengdu Normal University, Chengdu, Sichuan, China; 4 School of Computer and Software Engineering, Xihua University, Chengdu, Sichuan, China; Dayananda Sagar University, INDIA

## Abstract

The open radio access network (O-RAN) architecture facilitates intelligent radio resource management via RAN intelligent controllers (RICs). Deep reinforcement learning (DRL) algorithms are integrated into RICs to address dynamic O-RAN slicing challenges. However, DRL-based O-RAN slicing suffers from instability and performance degradation when deployed on unseen tasks. We propose M2DQN, a hybrid framework that combines multi-task learning (MTL) and meta-learning to optimize DQN initialization parameters for rapid adaptation. Our method decouples the DQN into two components: shared layers trained via MTL to capture cross-task representations, and task-specific layers optimized through meta-learning for efficient fine-tuning. Experiments in an open-source network slicing environment demonstrate that M2DQN outperforms MTL, meta-learning, and policy reuse baselines, achieving improved initial performance across 91 unseen tasks. This demonstrates an effective balance between transferability and adaptability. Code is available at: https://github.com/bszeng/M2DQN.

## Introduction

The evolution toward 6G networks introduce unprecedented complexity, driven by the coexistence of heterogeneous technologies and multi-band spectrum aggregation [1]. In this paradigm, optimization domains expand while network requirements become stricter, further complicating dynamic radio resource management (RRM) [2]. To address this challenge, open radio access network (O-RAN) architectures have emerged as critical enablers through their openness, which facilitates real-time data exposure, AI-driven optimization, and closed-loop control essential for adaptive RRM [3].

The O-RAN architecture empowers mobile operators (MNOs) with greater control over RRM by enabling data-driven closed-loop automation via machine learning (ML) for real-time network optimization and control. This is achieved by decoupling hardware and software through open interfaces [4]. The architecture includes two RAN Intelligent Controllers (RICs) that perform management and control of the network at near-real-time (10 ms to 1 s) and non-real-time (> 1 s) timescales, known as near-RT RIC and non-RT RIC, respectively [5].

Network slicing addresses 5G/6G’s heterogeneous QoS demands – enhanced mobile broadband (eMBB), massive machine-type communications (mMTC), and ultra-reliable low-latency communications (URLLC) – by logically partitioning physical infrastructure into virtual slices [6]. Each slice enforces strict service-level agreements (SLAs) compliance through dynamic resource allocation, adapting to time-varying traffic patterns.

Deep reinforcement learning (DRL) has emerged as a promising approach to address the complexities of O-RAN slicing, where traditional rule-based methods struggle with dynamic traffic patterns and multi-objective SLA constraints [7]. Unlike model-driven strategies, DRL algorithms leverage deep neural networks to adapt to evolving network conditions without requiring explicit formulations of stochastic wireless environments [8]. However, deploying DRL in real-world networks faces critical challenges, particularly in adapting to unseen network conditions. Such changes can destabilize DRL agents and degrade system performance, underscoring the importance of rapid convergence to optimal policies.

This paper addresses the critical challenge of enabling fast adaptation for DRL agents in O-RAN slicing. Our work focuses on mitigating instability and performance degradation, providing MNOs with efficient methods to accelerate DRL convergence and enhance overall network reliability. Our contributions can be summarized as follows:

- Propose a novel DQN initialization framework: We introduce a hybrid approach, M2DQN, that integrates MTL and meta-learning in non-RT RIC to initialize DQN in near-RT RIC. This method structures the DQN neural network into shared layers (updated through MTL) and task-specific layers (refined via meta-learning).
- Achieve an effective balanced integration of MTL and meta-learning: The shared layers capture common representations across multiple tasks, while the task-specific layers leverage these shared features for rapid adaptation. By fine-tuning the balance between shared and task-specific layers, the method achieves an effective integration, leveraging the strengths of both approaches.
- Validate the approach through comprehensive experiments: Extensive evaluations in an open-source network slicing environment demonstrate that our approach outperforms initialization strategies based solely on MTL, meta-learning, or policy reuse. This hybrid approach yields a highly generalizable DQN initialization strategy, improving initial performance and accelerating convergence across all unseen tasks.

The remainder of the paper is organized as follows: The Background section provides an overview of the problem, followed by a discussion of related work in the Literature section. The Problem Formulation section presents the system model. The Multi-task Meta-initialized DQN section describes the proposed hybrid approach and baseline methods. The Results section outlines the experimental setup and analyzes the results. Finally, the Conclusion and Future Work section concludes the paper and discusses future research directions.

## Background

### DRL for O-RAN slicing

Traditional methods such as queuing theory, Lagrange optimization, genetic algorithms, and heuristic approaches have been adopted for network slicing problems [9]. However, these methods fail to address the evolving requirements of next-generation cellular networks, particularly when handling constrained radio resources, dynamic channel conditions, multi-service interference, and heterogeneous QoS demands.

DRL techniques have recently emerged as a promising alternative for netwok slicing optimization. Unlike model-driven traditional approaches, DRL operates without a predefined network slicing model, instead learning optimal policies through direct environment interaction and iterative reward maximization [10]. In O-RAN architectures, DRL implementations are deployed across RIC layers: slicing xApps in near-RT RIC (to address radio-layer dynamics) and slicing rApps on the non-RT RIC (to orchestrate long-term strategies) [11].

**Fig 1:**
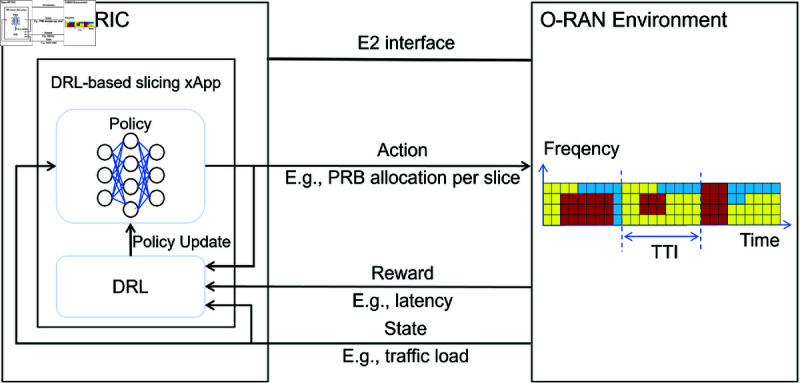
Architectural framework of DRL-based network slicing xApp integration within the O-RAN environment. Blocks of different colors represent PRBs allocated to different slices, with allocations updated at each transmission time interval (TTI).

As shown in Fig 1, the DRL framework consists of two primary components: The environment represents the O-RAN system state, while the agent (embodied in slicing xApps/rApps) observes network states and executes actions to maximize cumulative rewards. This closed-loop interaction enables continuous policy refinement, allowing DRL agents to dynamically adapt to fluctuating network conditions. The model-free nature of DRL eliminates dependency on analytical system models, making it particularly suited to managing the complex, non-stationary resource allocation challenges in next-generation cellular networks.

### Multi-task learning and meta-learning

Multi-task learning (MTL) demonstrates superior efficiency and effectiveness in learning shared representations by jointly utilizing training data from related tasks. Modern meta-learning extends this concept by using shared representations not only for joint training but also adapting rapidly to unseen tasks with minimal data during inference.

**Multi-task Learning**. MTL is an inductive transfer learning approach that harnesses the domain information from related tasks as inductive bias to improve generalization across all tasks [12]. Its core premise is that knowledge transfer between related tasks enhances model robustness beyond single-task learning [13]. By training tasks concurrently via parameter sharing, MTL implicitly encodes task correlations, a mechanism termed in [14].

Formally, consider *N* related tasks $𝒯=\{T_{1},T_{2},\ldots ,T_{N}\}$, where each task *T*_*i*_ has a training dataset $D_{i}^{train}$. MTL jointly optimizes shared parameters *θ* by minimizing:

$$\theta^{*}=\min_{\theta }\sum_{i=1}^{N}L_{i}(\theta_{i},D_{i}^{train})$$ (1)

where *L*_*i*_ denotes the task-specific loss. The aggregated loss promotes cross-task feature learning, yielding the optimal parameters $\theta^{*}$ that generalize effectively to unseen tasks.

**Meta-Learning**. Meta-learning ("learning to learn") [15] optimizes models for rapid adaptation to new tasks with limited data. Given *M* source tasks ${𝒯}_{s}=\{T_{s_{1}},T_{s_{2}},\ldots ,T_{s_{M}}\}$ from distribution p(𝒯), Meta-learning solves a bi-level optimization:

- **Inner Loop**. The inner learning algorithm solves a specific task defined by the training dataset $D_{s}^{train(i)}$ and its objective function *L^task^*. During task-specific learning, the optimal task-specific parameters $\theta^{*}$ are derived using the meta parameters *ϕ* as prior knowledge:
  $$\theta_{i}^{*}(\phi )=\arg\min_{\theta }L^{task}(D_{s}^{train(i)};\theta ,\phi )$$ (2)
- **Outer Loop**. The outer (meta) algorithm refines the inner learning process by optimizing the meta parameters *ϕ*. This optimization relies on unseen validation data $D_{s}^{val(i)}$, ensuring the model generalizes well to new tasks. The meta parameters *ϕ* are updated to minimize the meta loss *L^meta^* given the optimal task-specific parameters $\phi^{*}$:
  $$\phi^{*}=\arg\min_{\phi }\underset{T_{s}\sim p(T)}{𝔼}L^{meta}(D_{s}^{val(i)};\theta_{i}^{*}(\phi ),\phi )$$ (3)
  This process encodes knowledge from source tasks into the meta-parameters *ϕ*, enabling effective adaptation to new tasks. Here, *ϕ* represents the meta-parameters (also termed meta-knowledge), which encapsulate shared cross-tasks information to facilitate solving new ones.

**Synthesis for DRL Initialization**. While MTL and meta-learning differ mechanistically – MTL optimizes concurrent tasks versus meta-learning’s bi-level adaptation – both exploit task correlations to enhance generalization. Their outcomes ($\theta^{*}$ for MTL and $\phi^{*}$ for meta-learning) provide principled initialization schemes for DRL: MTL-derived $\theta^{*}$ encodes domain-invariant representations, while meta-trained $\phi^{*}$ preserves gradient pathways for rapid fine-tuning. By combining these strengths, DRL agents achieve accelerated convergence in dynamic network slicing scenarios.

## Literature

DRL-based applications to cellular network slicing have established foundational methodologies for dynamic resource orchestration under stochastic wireless environments. While existing works predominantly focus on traditional RAN architectures, the core theoretical frameworks exhibit significant applicability to O-RAN slicing scenarios [16].

DQN, a model-free DRL algorithm, has been successfully applied to solve network slicing problems, though it often requires extensive training steps [17]. Recent advancements like the federated-DQN slicing approach that offloads dynamic O-RAN disaggregation to edge nodes, enable localized data processing and faster decision-making [18]. A DQN-based O-RAN slicing approach learns control policies under varying SLAs with heterogeneous minimum performance requirements [19]. Mhatre *et al.* propose a DQN-based strategy for QoS-aware intra-slice resource allocation, optimizing for eMBB and URLLC slices [20]. DQN is introduced through an adaptive standardized protocol to address inter-slice resource contention and conflict in network slicing [21].

However, DQN-based solutions suffer from instability and performance degradation when deployed on unseen tasks. While architectural refinements have improved convergence [22], enhancing adaptability to unseen tasks requires integrating transfer learning strategies such as transfer learning(TL), MTL, and meta-learning [23].

TL accelerates DRL adaptation by reusing knowledge from related source tasks [24]. Nagib *et al.* demonstrated policy transfer via initializing new agents with pre-trained policies [25]. Despite its benefits, TL requires mechanisms to evaluate task transferability and mitigate negative transfer caused by domain mismatches [26]. These limitations highlight the need for more robust cross-task generalization frameworks.

Multi-task learning (MTL) addresses these challenges by sharing knowledge across tasks during joint training. Liu *et al.* [27] proposed an MTL-based resource allocation algorithm for multi-objective optimization, lowering computational costs. Lei *et al.* [28] developed a multi-task DRL framework to exploit task commonalities and differences. Dong *et al.* [29] further applied MTL to simplify action spaces in fine-grained network slicing, and Gracla *et al.* [30] enhanced robustness against data distribution shifts using MTL-based DRL.

In parallel, meta-learning focuses on rapid adaptation for unseen tasks with minimal data. Yuan *et al.* [31] designed a meta-DRL algorithm for dynamic V2X resource allocation, and later Yuan *et al.* [32] presented a meta-DRL algorithm to adapt quickly to dynamic environmental changes in wireless networks. Hu *et al.* [33] demonstrated meta-learning’s value in drone base station control, accelerating DRL convergence for optimal coverage.

While MTL improves training efficiency across heterogeneous tasks and meta-learning enables fast adaptation, their integration remains underexplored. [34]. Theoretical studies suggest combining these paradigms could reduce training steps while boosting performance on unseen tasks [35]. Despite these advantages, existing efforts offer limited insights into combining MTL and meta-learning within DRL, particularly in accelerating adaptation to unseen O-RAN slicing tasks.

To bridge this gap, we propose M2DQN – a novel DQN initialization paradigm that hierarchically combines MTL (for shared layers) and meta-learning (for task-specific layers). By optimizing neural network parameters during pretraining, M2DQN enhances adaptability to deployment-specific unseen scenarios.

## Problem formulation

### System model

In network slicing optimization, MNOs dynamically adjust SLA priorities by tuning KPI weights in the DRL reward function per network slice [25]. This paper specifically addresses downlink-oriented network slicing scenarios, focusing on flexible allocation of limited physical resource blocks (PRBs) while maintaining target thresholds for spectral efficiency, latency, and quality of experience.

Following the formulation in [36], we consider *S* network slices sharing total bandwidth *B*, with PRB allocation represented by vector $x\in {\mathbb{R}}^{S}$. The O-RAN slicing controller selects allocation configuration *x*(*a*) from *X* possible configurations (a=1,2,3,…,X), where each selection significantly impacts system performance.

System performance is quantified through aggregated slice latency metrics:

f(x(a),o(t))=αL∈ℝ, (4)

where *L* denotes the inverse latency measure, *α* represents a slice-specific latency weighting coefficient, and *o*(*t*) captures the time-varying system state at time *t*. The system state *o*(*t*) evolves under the influence of dynamic network parameters including traffic load, channel quality, and external environmental disturbances. Many of these parameters exhibit non-stationary behavior that resists analytical modeling, particularly at sub-second timescales. To address this complexity, the O-RAN slicing controller explores various slice allocation configurations and evaluates their corresponding impacts on system performance. This process continues until discovering the optimal configuration that maximizes overall system performance metrics.

### Deep Q-network

Deep Q-Network (DQN), a model-free DRL algorithm, has been widely adopted for network slicing [7]. By integrating neural networks into the Q-learning framework, DQN approximates the state-value function to enable data-driven decision-making. The learning process is structured through three core components:

- **States**. The state o∈O (illustrated in Fig 1), observed by the DRL agent (the slicing xApp), integrates dynamic network parameters including per-slice traffic load metrics, channel quality, and other external variables affecting the performance of the O-RAN system.
- **Actions**. At each slicing window onset, the agent selects an action based on the observed state. The selected action a∈𝒜 specifies resource allocation across slices, where 𝒜 represents the feasible action space with cardinality |𝒜|. This decision is mathematically formulated as a bandwidth proportion vector:
  $$a=(b_{1},b_{2},\ldots ,b_{S}),\text{ subject to }b_{1}+b_{2}+\ldots +b_{S}=B$$ (5)
- **Rewards**. The network generates reward signals after the agent executes action *a* in state *o*, triggering a state transition to $o^{\prime }$. Network engineers design this reward function as a weighted combination of KPIs to guide policy optimization. Specifically, we formulate the DRL reward *R* using parametric sigmoid transformations of slice latencies:
  $$R=\sum_{s=1}^{\left\|S\right\|}w_{s}\times \frac{1}{1+e^{c1_{s}\times (l_{s}-c2_{s})}},$$ (6)
  Here, *s* indexes slices, *l*_*s*_ represents latency, and *w*_*s*_ denotes the importance of latency for each slice. The parameters *c*1 and *c*2 are configured for each slice type to adjust the shape of the sigmoid function, thereby determining when and how latency violations penalize the agent’s actions. Parameter *c*1 defines the point at which the slope of the sigmoid function begins to change, indicating when to start penalizing the agent’s actions. Meanwhile, *c*2 represents the inflection point - the minimum acceptable delay performance for a slice, as determined by its SLAs. To account for the unique requirements of different slice types, distinct but constant *c*1 and *c*2 values are assigned to each slice type.
- **Q-function**. The state-value function, or Q-function, uses a neural network to estimate the Q-value, denoted as *Q*(*o*,*a*), which represents the expected cumulative reward obtained after taking action *a* in state *o*.

At each slicing window initialization, the DRL agent decides PRB allocation *a* based on observed system states *o*, aiming to maximize long-term expected rewards through dynamic resource optimization. Formally:

$$\arg\min_{a}\;𝔼\{R(o,a)\}\;\text{subject to }b_{1}+b_{2}+\ldots +b_{S}=B,\;a=\pi (o)\in A\;$$ (7)

where *π* denotes a policy function that maps the system state *o* to an action *a* within the feasible action space *A*, with 𝔼(·) representing the expectation operator. The primary challenge in solving Eq (7) lies in handling time-varying demands, traffic model variations, and fluctuating user numbers across different service types. While an exhaustive search for optimal solutions is theoretically possible, it is computationally infeasible. Therefore, DRL provides a practical approach to address this dynamic allocation problem.

## Multi-task meta-initialized DQN

This section investigates a DRL environment designed for O-RAN slicing adaptation. In this scenario, source network slicing tasks serve as training environments where the DRL agent acquires transferable policy knowledge. Upon completing training, the agent deploys to handle unseen slicing tasks characterized by novel configurations in the DRL reward function. Specifically, each task is uniquely defined through weighting coefficients $\{w_{s}{\}}_{s=1}^{S}$ assigned to slice-specific KPIs. When MNOs introduce target tasks with previously unseen reward function weights, the pre-trained agent demonstrates rapid policy convergence, enabling efficient adaptation to the new network conditions.

**Fig 2:**
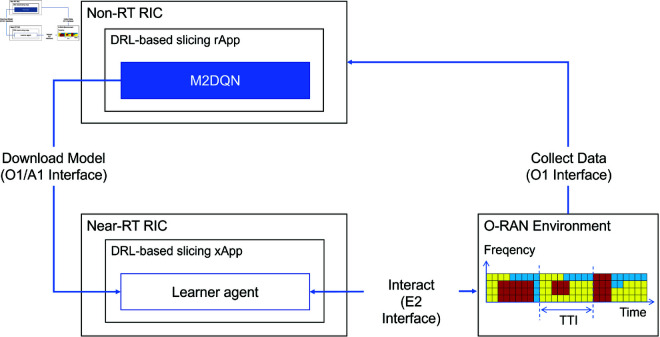
M2DQN workflow in RICs. M2DQN operates as a trained non-RT RIC rApp. The learner agent in the near-RT RIC downloads initialization parameters from M2DQN when encountering new slicing tasks.

As depicted in Fig 2, the M2DQN architecture operates across O-RAN’s RIC components. The non-RT RIC hosts M2DQN (blue module) as an rApp that learns DQN initialization parameters through training using historical data collected via the O1 interface. The near-RT RIC deploys a DRL-based slicing xApp ("learner agent"), which loads M2DQN-trained parameters through the AI interface and executes real-time control via the E2 interface, as explicitly shown in the figure’s dataflow.

**Fig 3:**
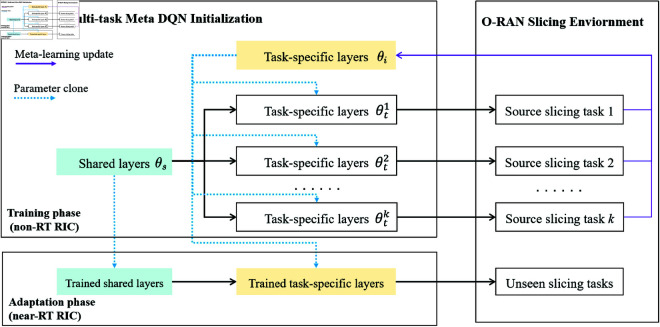
Architecture of M2DQN. The shared layer parameters $\theta_{s}$ are continuously updated across all source tasks. For each source task, the task-specific layer parameters $\theta_{i}$ are individually optimized based on the trained parameters $\theta_{t}$. During adaptation, the pre-trained $\theta_{s}$ and $\theta_{i}$ initialize the shared and task-specific layers, respectively, enabling rapid adaptation of unseen tasks.

Fig 3 depicts the principal structure of M2DQN with two core components: shared layers and task-specific layers. The architecture employs a bi-level optimization strategy: Shared layers are trained via MTL through blue dashed arrows to capture cross-task representations, while task-specific layers are updated via purple solid arrow updated via meta-learning to adapt shared features. During training, both layers are jointly updated through solid-line connections, with additional task-specific parameter refinements applied post-task to address initialization deviations. In adaptation, the trained parameters of both the shared and task-specific layers are duplicated and deployed for use in unseen slicing tasks.

**Algorithm 1 Multi-task meta-initialized DQN.**

Initialize the parameters $\theta_{s}$ (shared layers) and $\theta_{i}$ (task-specific layers).

Initialize target networks: $\theta_{s}^{-}=\theta_{s}$ and $\theta_{i}^{-}=\theta_{i}$.

Initialize temporary parameters $\theta_{t}$ and target $\theta_{t}^{-}$.

1: **for** each source slicing task **do**

2:   $\theta_{t}\leftarrow \theta_{i}$, $\theta_{t}^{-}\leftarrow \theta_{i}$.

3:   **for** each episode **do**

4:    **for** step *t* to the end of episode **do**

5:     Observe state *o*_*t*_.

6:     Select action *b*_*t*_ via *ε*-greedy policy.

7:     Execute *b*_*t*_, obtain reward *r*_*t*_ and next state *o*_*t* + 1_.

8:     Store transition $\left(o_{t},b_{t},r_{t},o_{t+1}\right)$ in buffer ℬ.

9:     Sample a mini-batch of transitions from ℬ.

10:     Compute time difference loss $L(\theta_{s},\theta_{t})$ via Eq (8).

11:     Update $\theta_{s}$, $\theta_{t}$ via Eq (9).

12:     **if** t mod $N_{N}^{-}==0$ **then**

13:      Sync target networks: $\theta_{s}^{-}\leftarrow \theta_{s}$ and $\theta_{t}^{-}\leftarrow \theta_{t}$.

14:     **end if**

15:    **end for**

16:   **end for**

17:   Update $\theta_{i}$ by Eq (10).

18: **end for**

19: Output ${\hat{\theta }}_{s}=\theta_{s}$ and ${\hat{\theta }}_{i}=\theta_{i}$.

Algorithm 1 provides the M2DQN framework’s pseudocode, initializing shared layer parameters $\theta_{s}$ and task-specific layer parameters $\theta_{i}$ randomly, with target networks $\theta_{s}^{-}\leftarrow \theta_{s}$ and $\theta_{i}^{-}\leftarrow \theta_{i}$. At the start of each source task, the temporary parameters $\theta_{t}$ are cloned from $\theta_{i}$ and updated throughout the task. The agent selects an action *b*_*t*_ at step *t* using the *ε*-greedy strategy, where random exploration occurs with probability *ε*, and the optimal action is chosen with probability 1−ε. The agent then interacts with the O-RAN slicing environment by executing action *b*_*t*_, observing the resulting next state *o*_*t* + 1_ and reward *r*_*t*_. This interaction generates the experience $\left(o_{t},b_{t},r_{t},o_{t+1}\right)$, which is stored in the replay buffer ℬ.

During each step, a mini-batch of transitions is sampled from ℬ to minimize the loss function L(θ):

$$L(\theta )=(r+\gamma \max_{a\in A}Q(o^{\prime },a;\theta^{-})-Q(o,a;\theta ){)}^{2}$$ (8)

where γ∈[0,1] is the discount factor. The parameters $\theta =\{\theta_{s},\theta_{t}\}$ define the DQN network, while $\theta^{-}=\{\theta_{s}^{-},\theta_{t}^{-}\}$ represent the target network parameters. *θ* is updated via gradient descent:

$$\theta \leftarrow \theta -\eta \nabla_{\theta }L(\theta )$$ (9)

Every $N_{N}^{-}$ steps, $\theta_{s}$ and $\theta_{t}$ are synchronized with $\theta_{s}^{-}$ and $\theta_{t}^{-}$, respectively. Upon task completion, the trained temporary task-specific parameter $\theta_{t}^{*}$ updates the task-specific parameter $\theta_{i}$ via Eq 10 in a Reptile-style meta-learning framework [37]:

$$\theta_{i}\leftarrow \theta_{i}+\lambda (\theta_{t}^{*}-\theta_{i})$$ (10)

where *λ* is the meta-learning rate. Upon completing all episodes of a task, the task-specific parameter $\theta_{i}$ is updated via Eq (10) before transitioning to the next task. At the initiation of a new task, the temporary parameter $\theta_{t}$ is cloned from $\theta_{i}$. The shared parameters $\theta_{s}$ undergo continuous gradient-based updates at every step, whereas $\theta_{i}$ is updated once per task. This hybrid strategy integrates MTL and meta-learning: MTL for $\theta_{s}$ (continuous per-step updates) and meta-learning for $\theta_{i}$ (per-task adjustments). The trained parameters $\theta_{s}$ and $\theta_{i}$ are stored as ${\hat{\theta }}_{s}$ and ${\hat{\theta }}_{i}$, respectively. When deployed to new tasks, ${\hat{\theta }}_{s}$ and ${\hat{\theta }}_{i}$ initialize the learner agent’s networks, and the agent updates the network parameters by following the standard DQN process.

## Results

We conduct simulations in an open-source network slicing environment supporting three types of services: VoLTE, URLLC, and video. User requests are generated following the statistical distributions defined in Table 1. To evaluate the generalizability of DRL initialization algorithms, we train these models on a limited set of network slicing scenarios. The trained models are subsequently deployed and tested on unseen scenarios. Various network slicing scenarios are generated by modifying the reward function weights within the environment.

**Table 1:** Network slicing parameter settings.

Parameters	VoLTE	URLLC	Video
Scheduling	Round Robin per 0.5 ms slot		
Bandwidth allocation window size	2000 scheduling time slots (1 second)		
Packet interarrival time distribution	Truncated Pareto	Uniform	Exponential
	Min: 0ms, Max: 160ms	Mean: 180ms	Mean: 6ms, Max: 12.5ms
Packet size distribution	Uniform	Truncated Lognormal	Truncated Pareto
	40Byte	Mean: 2MB, Std.: 0.722MB, Max: 5MB	Mean: 100Byte, Max: 250Byte

Following the weight combination settings from [25], we employ 16 source tasks for model training and 91 unseen weight configurations (excluded during training) to assess adaptability, as detailed in Table 2. Each combination reflects the emphasis on different slices: for example, [0.1, 0.1, 0.8] shows extreme bias towards Video, [0.1, 0.4, 0.5] indicates dominance of Video and URLLC, and [0.33, 0.33, 0.33] represents complete equally balanced priorities. MNOs can dynamically adjust weight combinations according to real-time traffic patterns [38].

**Table 2:** Reward function weight combinations.

Simulation stage	Dominant priorities			Balanced	Hybrid priorities		
	VoLTE	URLLC	Video	priorities	VoLTE + URLLC	VoLTE + Video	URLLC + Video
Training	[0.8,0.1,0.1]	[0.1,0.1,0.8]	[0.1,0.1,0.8]	[0.4,0.3,0.3]	[0.4,0.5,0.1]	[0.4,0.2,0.4]	[0.1,0.5,0.4]
		[0.1,0.7,0.2]	[0.1,0.2,0.7]	[0.33,0.33,0.33]	[0.4,0.4,0.2]	[0.4,0.1,0.5]	[0.1,0.4,0.5]
		[0.1,0.6,0.3]	[0.1,0.3,0.6]				[0.1,0.45,0.45]
Adaptation	[0.5,0.25,0.25]	[0.1,0.75,0.15]	[0.1,0.15,0.75]	[0.3,0.3,0.4]	[0.3,0.45,0.25]	[0.3,0.1,0.6]	[0.1,0.35,0.55]
	[0.6,0.2,0.2]	[0.1,0.85,0.05]	[0.1,0.25,0.65]	[0.3,0.35,0.35]	[0.3,0.5,0.2]	[0.3,0.15,0.55]	[0.1,0.55,0.35]
	[0.6,0.25,0.15]	[0.15,0.6,0.25]	[0.15,0.1,0.75]	[0.3,0.4,0.3]	[0.3,0.55,0.15]	[0.3,0.2,0.5]	[0.1,0.65,0.25]
	[0.65,0.1,0.25]	[0.15,0.7,0.15]	[0.15,0.2,0.65]	[0.35,0.3,0.35]	[0.3,0.6,0.1]	[0.3,0.25,0.45]	[0.15,0.3,0.55]
	[0.65,0.2,0.15]	[0.15,0.8,0.05]	[0.2,0.1,0.7]	[0.35,0.4,0.25]	[0.35,0.5,0.15]	[0.35,0.1,0.55]	[0.15,0.4,0.45]
	[0.7,0.1,0.2]	[0.2,0.6,0.2]	[0.2,0.15,0.65]	[0.4,0.35,0.25]	[0.35,0.6,0.05]	[0.35,0.2,0.45]	[0.15,0.5,0.35]
	[0.7,0.15,0.15]	[0.2,0.7,0.1]	[0.2,0.2,0.6]	[0.25,0.4,0.35]	[0.4,0.45,0.15]	[0.4,0.15,0.45]	[0.2,0.3,0.5]
	[0.7,0.2,0.1]	[0.2,0.75,0.05]	[0.2,0.25,0.55]	[0.4,0.25,0.35]	[0.4,0.55,0.05]	[0.45,0.1,0.45]	[0.2,0.35,0.45]
	[0.7,0.25,0.05]	[0.25,0.5,0.25]	[0.25,0.1,0.65]		[0.45,0.3,0.25]	[0.45,0.2,0.35]	[0.2,0.4,0.4]
	[0.75,0.1,0.15]	[0.25,0.7,0.05]	[0.25,0.2,0.55]		[0.45,0.4,0.15]	[0.5,0.1,0.4]	[0.2,0.45,0.35]
	[0.75,0.2,0.05]	[0.3,0.65,0.05]			[0.45,0.45,0.05]	[0.5,0.15,0.35]	[0.2,0.5,0.3]
	[0.8,0.15,0.05]				[0.5,0.25,0.15]	[0.5,0.2,0.3]	[0.2,0.55,0.25]
	[0.85,0.1,0.05]				[0.5,0.3,0.2]	[0.55,0.1,0.35]	[0.25,0.3,0.45]
					[0.5,0.4,0.1]	[0.55,0.2,0.25]	
					[0.5,0.45,0.05]	[0.6,0.1,0.3]	
					[0.55,0.3,0.15]	[0.6,0.15,0.25]	
					[0.55,0.4,0.05]		
					[0.6,0.3,0.1]		
					[0.6,0.35,0.05]		
					[0.65,0.3,0.05]		

We adopt three DQN variants for policy initialization: Multi-task DQN, Meta-learning, and M2DQN. As detailed in Table 3, all approaches share a three-layer neural architecture with (32, 32, 15) neurons per layer but diverge in their update mechanisms:

- Multi-task DQN: Optimizes all layers through MTL.
- Meta DQN: Updates layers purely via meta-learning.
- M2DQN: Hierarchically integrates MTL for shared layers and meta-learning for task-specific layers.

**Table 3:** DRL architecture parameters.

Model	First layer:32	Hidden layer:32	Last layer:15
Multi-task DQN	MTL	MTL	MTL
Meta DQN	meta-learning	meta-learning	meta-learning
M2DQN	MTL	meta-learning	meta-learning

The specific network slicing parameters are provided in Table 4.

**Table 4:** RAN slicing DRL parameters.

Parameters	Value
State	($d_{\text{VoLTE}},d_{\text{URLLC}},d_{\text{Video}}$)
Action	15 allocation configurations
Reward	$c1_{\text{VoLTE}}$: 0.5, $c1_{\text{URLLC}}$: 2, $c1_{\text{Video}}$: 1, $c2_{\text{VoLTE}}$: 10, $c2_{\text{URLLC}}$: 3, $c2_{\text{Video}}$: 7
Max episode timesteps	100
Total episodes	50
Number of adaptation tasks	1729 (91×19)

To evaluate adaptability, we pre-train 19 agents (16 for source tasks, 3 for proposed approaches) using distinct weight configurations from Table 2. These agents are applied to 91 unseen tasks through policy transfer, generating 1,729 adaptation tasks. All algorithms and configurations are implemented and publicly available on https://github.com/bszeng/M2DQN.

We evaluate the performance enhancement of accelerated DQN algorithms - policy reuse [25], Multi-task DQN, Meta-DQN, and M2DQN - for initializing agents across 91 unseen tasks. Policy reuse trains agents via a DQN on a single source task, while Multi-task DQN, Meta-DQN, and M2DQN leverage 16 source tasks. Statistical improvements in reward after the first episode are summarized in Table 5.

**Table 5:** Accelerated algorithms adaptation statistics.

Accelerated algorithms	Degraded	Improved	Accelerated algorithms	Degraded	Improved
Policy reuse by [0.8,0.1,0.1]	12	79	Policy reuse by [0.1,0.45,0.45]	50	41
Policy reuse by [0.4,0.2,0.4]	12	79	Policy reuse by [0.1,0.2,0.7]	50	41
Policy reuse by [0.4,0.5,0.1]	17	74	Policy reuse by [0.4,0.3,0.3]	58	33
Policy reuse by [0.4,0.1,0.5]	17	74	Policy reuse by [0.1,0.4,0.5]	58	33
Policy reuse by [0.4,0.4,0.2]	47	44	Policy reuse by [0.1,0.8,0.1]	58	33
Policy reuse by [0.1,0.5,0.4]	47	44	Policy reuse by [0.33,0.33,0.33]	58	33
Policy reuse by [0.1,0.6,0.3]	47	44	**Meta DQN**	**58**	**33**
Policy reuse by [0.1,0.7,0.2]	47	44	**Multi-task DQN**	**3**	**88**
Policy reuse by [0.1,0.1,0.8]	50	41	**M2DQN**	**0**	**91**
Policy reuse by [0.1,0.3,0.6]	50	41			

M2DQN consistently outperforms all baselines, achieving improvements across all 91 unseen tasks. Multi-task DQN ranks second but fails to improve performance in three tasks. Notably, among the 16 policy reuse configurations, weight settings [0.8,0.1,0.1] and [0.4,0.2,0.4] deliver the best performance, enhancing initial performance in 79 out of 91 tasks. These results validate M2DQN’s superior adaptability and generalization.

The findings highlight two critical advantages of M2DQN and Multi-task DQN. First, both algorithms learn transferable initialization parameters that enhance adaptability to dynamic slicing conditions. Second, unlike policy reuse, they reduce the need for exhaustive source-target task relationship analysis, reducing the deployment complexity of the DQN algorithm.

**Fig 4:**
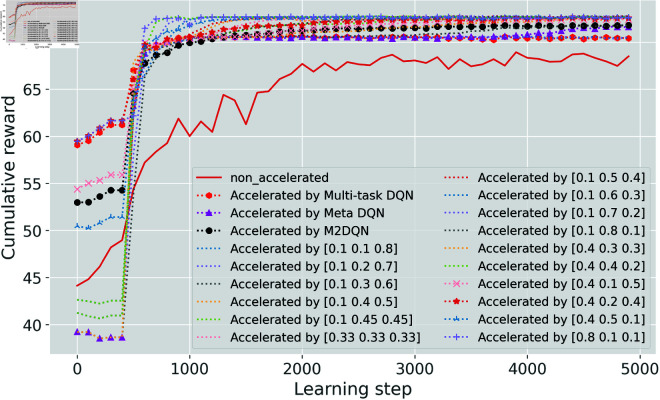
Cumulative reward. Accelerated algorithms may initially underperform baseline methods during early learning stages, but demonstrate superior convergence performance.

The convergence behavior of average rewards for unseen tasks is depicted in Fig 4 when trained agents are used to initialize new agents. The solid red line in the figure represents the average reward across 91 unseen tasks without initialization using trained agents. Each of the other lines represents the average reward across 91 unseen tasks initialized with one trained agent. Six accelerated DQN algorithms, including M2DQN, Multi-task DQN, and four TL-based algorithms, exhibit improved performance in the early stages of learning.

**Fig 5:**
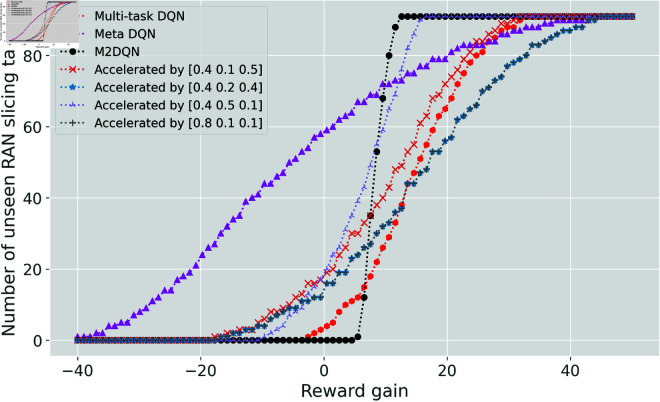
Initial reward gain comparison. A positive value indicates that the reward achieved in the first episode by the accelerated algorithm exceeds that of the non-accelerated algorithm. A larger value signifies greater improvement, whereas a negative value indicates a decline.

For a detailed examination of these six enhanced agents, Fig 5 plots the cumulative distribution function of the first-episode reward gain. The horizontal axis quantifies the reward gain relative to the non-accelerated algorithms, while the vertical axis indicates the number of unseen tasks achieving each reward gain level.

An analysis of the two graphs in Fig 5 reveals that the initial rewards of M2DQN (represented by the black dashed line with circle markers) are concentrated between 5 and 20. This clustering indicates that M2DQN effectively learns initialization parameters with strong generalization capabilities for DQN-based systems. Although M2DQN does not always achieve the highest initial performance on unseen tasks, its performance shows no degradation – a critical requirement for MNOs. Overall, M2DQN achieves an effective balance between adaptation capability and task performance.

## Conclusion and future work

This paper presents M2DQN – a hybrid DRL initialization framework that synergizes multi-task learning and meta-learning to enhance DQN adaptability in O-RAN slicing. Deployed in the non-RT RIC, M2DQN employs shared layers to extract cross-task representations from 16 source tasks, while its task-specific layers enable rapid fine-tuning via meta-learning. Experimental results in an open-source network slicing environment demonstrate that M2DQN effectively learns transferable initialization parameters from 16 source tasks and achieves performance improvements across all 91 unseen tasks. This work advances DQN generalizability by addressing the critical challenge of fast adaptation in dynamic network slicing scenarios.

Moving forward, our research will focus on two synergistic directions to enhance dynamic network slicing adaptability: 1) Intelligent Task Selection, which improves training efficiency through posterior and diversity-based task sampling; and 2) Regularization-Enhanced Generalization, which integrates adaptive dropout mechanisms to mitigate overfitting risks. By coupling these directions, the dual-pronged approach aims to simultaneously reduce adaptation latency and suppress performance degradation caused by overfitting.
